# Navigating access barriers: Strategies for Syrian refugees confronting healthcare challenges in Jordan - A qualitative approach

**DOI:** 10.1371/journal.pgph.0006136

**Published:** 2026-05-04

**Authors:** Ibraheem Khaled Abu-Siam, María Rubio Gómez, Bahaa Ahmad Alassoud, Ahmad Salim Batran, Ashraf Isamil Alamery

**Affiliations:** 1 Migration Institute, Granada University, Granada, Spain; 2 Faculty of Nursing, Palestine Ahliya University, Bethlehem, Palestine; 3 Public Health Unit, United Nations High Commissioner for Refugees, Amman, Jordan; Keele University, UNITED KINGDOM OF GREAT BRITAIN AND NORTHERN IRELAND

## Abstract

The Syrian refugee crisis represents one of the most significant instances of forced displacement in recent history. Hosting approximately one-third of all Syrian refugees in the Middle East, Jordan faces considerable strain on its healthcare system. This qualitative descriptive study explores the strategies Syrian refugees in Jordan employ to confront access barriers when healthcare is needed. The data was collected in late 2019 from twenty participants through in-depth interviews. Purposive sampling with maximum variation was utilized until thematic saturation was reached. The data was analyzed using Braun and Clarke’s reflexive thematic analysis. Of the 20 participants, the majority (60%) were male, and all had a low level of formal education. Most families (90%) were male-headed, with an average family size of 5.1 members. Our analysis identified ten key themes, grouped into two categories. The first category encompasses health-related adaptation strategies, including self-medication, using alternative medicine, delaying care, stopping or reducing medication, and seeking free health services. The second category involves non-health related strategies, in which health needs were reprioritized over other livelihood essentials. These included borrowing money, collecting donations, resorting to illegal child labor, and planning to relocate to other countries. This study has unveiled a range of adaptation strategies employed by Syrian refugees to overcome access barriers to essential healthcare services. These findings carry important implications for healthcare practice and policy, offering insights that can be used to enhance refugees’ overall well-being and ensure sustainable, equitable, and coordinated access to healthcare services. By understanding these strategies, policymakers and healthcare providers can design targeted interventions aimed at improving healthcare accessibility for this vulnerable population.

## 1 Introduction

The global mobility of human beings is a growing concern in an increasingly volatile world. As per the United Nations High Commissioner for Refugees (UNHCR) records, the number of displaced people globally had tripled between 2010 and 2024 and hit the highest-ever recorded number at 123.2 million individuals [1]. According to the latest data, there are 31 million refugees worldwide, with the majority residing in Asia (17.3 million) and Europe (13.2 million). The 2011 onset of civil war in Syria (the Syrian crisis), has led to one of the most significant refugee situations in recent history with approximately 6.28 million Syrian refugees fleeing the country and registering with the UNHCR by 2024 [1]. Currently, Syria contributes to about 20% of the global refugee load [1].

The Middle East and North Africa (MENA) region hosts approximately 2.3 million refugees and is considered the fourth-highest refugee zone globally [1]. The MENA region has witnessed a series of conflicts over the last 25 years resulting in refugee crises, these include Iraq in 2003, Syria in 2011, and Yemen in 2014, to Sudan in 2019. These conflicts have led to massive movements of people either within their own country (Internally Displaced Persons (IDPs)) or crossed internationally recognized borders and become a refugee across the region.

Historically, Jordan has been home to a diverse refugee population, hosting over three-quarters of a million people predominantly from Syria, Iraq, Yemen, and Sudan [2]. It is one of the most affected countries in this region. Currently, Jordan hosts more than 675,000 refugees, the majority of whom reside in urban sites (81.4%) [3]. Syrian refugees alone account for 89. 1% of the total refugee load in the country, with approximately 611,000 refugees registered in the UNHCR database [3].

Syrian refugees hosted in camps rely on humanitarian assistance programs, covering shelter, water, food, education, health care and other livelihoods. In contrast, most urban refugees meet their needs for essential services within the existing urban system. The Government of Jordan has supported refugees’ access to essential services, including civil registration, the justice system, the labor market, free essential education, and subsidized health care through public healthcare systems and structures.

### 1.1 Overview of health services and access policy for Syrian refugees in Jordan

Ministry of Health (MoH) is the overall umbrella for all health affairs in Jordan [4]. It is one of the major providers of preventive health services, curative care, and health insurance for Jordanians. Jordan’s healthcare system is a mixed model system with public, private, and humanitarian providers sharing the responsibilities for service provision and complementing one another. By the end of 2019, total expenditure on healthcare reached 3.1 billion USD, accounting for 7.47% of the country’s GDP [5].

Since the onset of Syrian crisis in 2011, Jordan has provided primary healthcare services to Syrian refugees. As part of its public health strategy,the Government of Jordan developed and adopted several healthcare policies to facilitate access to Syrian refugees. In March 2012, the government allowed urban registered Syrian refugees to access MoH health centers and hospitals free of charge. This policy remained in effect until November 2014. At that time, the copayment modality was introduced, requiring refugees to contribute to the cost of their treatment [6].

In January 2018, the government adopted a new policy that increased the copayment for healthcare services fourfold. However, in January 2019, the government reversed this decision, reinstating the old healthcare access policy. Throughout these policy changes, the government maintained an important exception for all services under the expanded programme on immunization (EPI), ensuring they remained free of charge to all children and pregnant women.

### 1.2 Barriers to health care among refugees

Barriers are broadly defined as “anything used or acting to block someone from going somewhere or doing something or to block something from happening” [7]. In the context of healthcare, several theoretical models have conceptualized access barriers. For instance, Andersen’s behavioral model established large scale barriers including demographic variables, social structure such as education, occupation, family structure, and beliefs that influence life style, physical environment and health seeking behaviors. Similarly, the Healthcare Access Barriers (HCAB) model provides a framework for evaluating modifiable and non-modifiable barriers, classifying them into structural, cognitive and financial barriers [8].

Current available evidence consistently demonstrates that mobile populations, including asylum seekers, regularly face difficulties in accessing or utilizing healthcare in the country of asylum [9,10]. The association between refugees’ status and healthcare access barriers is well established. For instance, cognitive, structural, socio-political, and financial barriers have been identified as major categories of barriers that refugees may encounter in different contexts [11]. Several researchers have identified individualized healthcare access barriers across various refugee contexts [12]. While some of these barriers are prevalent among refugees in Jordan, others appear to be less significant in this specific setting. For example, socio-political barriers such as legal status and language, commonly reported in other contexts are not major obstacles for refugees in Jordan. This is largely due to the clear legal recognition of refugees and the cultural and linguistic compatibility between Syrian refugees and the host population.

Contrariwise, other barriers frequently reported in global literature, such as poor disease perception, long waiting times, limited knowledge of available services, geographic distance, and cost of care are consistently observed among Syrian refugees in Jordan ^[^13–16^]^. These findings highlight the importance of contextualizing healthcare access barriers, as their relevance and impact may vary significantly depending on the refugees background, refuge journey, conflict nature or health system structure and sociopolitical environment of the host country. In summary, access barriers among displaced populations have been extensively investigated in several contexts and population groups. The set of identified access barriers varies depending on study context and have been classified in several ways by several health models. Collectively, what has been identified represents a wide spectrum of barriers that can affect any mobile population in different settings, underscoring the importance of considering context specific conditions when analyzing access difficulties. A nuanced understanding of local conditions is key to designing health interventions that improve care access within its unique environment.

### 1.3 Current study

Facing barriers to healthcare during refuge journeys is a common challenge faced widely, often due to a combination of structural, cognitive, and socio-political factors [17]. However, it is equally important to explore how refugees respond when facing prolonged limitations in accessing healthcare. This study aims to explore the strategies adopted by Syrian refugees in Jordan to address their long-term healthcare needs after several years of living in unstable conditions.

## 2 Methods

### 2.1 Ethics statement

Participants who agreed to take part in the study received an information sheet in Arabic, and verbal informed consent was obtained for both participation and audio recording. Interviews were conducted between November 2019 and January 2020. The study protocol received ethical approval from the Jordan Health Sector Research Committee of UNHCR. Interviews were conducted, transcribed and analyzed in Arabic to ensure accuracy and preserve contextual meaning. To protect participants’ confidentiality, all transcripts were de-identified, and pseudonyms were assigned to replace real names or any identifiable personal information. All audio files, transcripts, and translated documents were stored in password-protected and encrypted digital folders accessible only to the research team.

Field notes were recorded throughout the data collection phase, including reflections on assumptions and anticipated outcomes. The potential influence of the researcher’s background and prior knowledge was acknowledged and regularly assessed for its impact on the study findings. This ongoing reflexivity prompted regular re-evaluation during data collection to identify and minimize potential biases.

### 2.2 Design

With limited availability of predetermined information in this area and limited opportunities given for this vulnerable group to voice their experiences, the open qualitative descriptive design was employed to provide a comprehensive in-depth perspective on potential adaptations among refugees who have experienced long term access barriers [18]. As outlined by Doyle and others, this design emphasizes flexibility and adaptability, allowing emergent themes to guide inquiry without predefined hypotheses [19]. This approach creates a space to understand the associated phenomena and strategies used to address long term health needs. Additionally, Kim and his team highlighted the unique characteristic of this design in prioritizing participants’ narratives over unnecessary “methodological acrobatics” [20] of which will help to explore the new insights grounded in the participants lived experiences and perspectives.

### 2.3 Settings

Participants who agreed to take part in the study were interviewed in specially designated place within three community centers located in Amman and Zarqa. These two cities are major urban centers in Jordan and host the largest and most diverse urban refugees. By selecting community centers as interview sites, the study aimed to capture a wide range of experiences and perspectives, regardless of participants’ access status to healthcare services. These settings are often more familiar and refugee-friendly than clinical settings, encouraging more open, honest and non-stigmatized participation.

### 2.4 Participants

Twenty participants were purposively selected for the qualitative phase of the doctoral study [21] from a larger group of 380 participants who were selected using simple random sampling technique, including those who were of Syrian nationality registered outside officially recognized refugee camps, above the age of 18 and were either the head of the HH or a knowledgeable caregiver living in the same HH. From this pool, participants were chosen based on their willingness to continue with the study, their ability to express themselves clearly, and their demonstrated understanding of their family’s health situation. Selection for the qualitative assessment was conducted without gender preference.

Theoretical saturation was reached at 18 interviews, when no new information was generated from ongoing data collection. However, two additional interviews were conducted to ensure sufficient geographical variation across the study sites.

### 2.5 Data collection

Data collection involved semi-structured interviews, striking a balance between methodological robustness and the flexibility to capture refugees’ complex and diverse experiences in a scientifically rigorous and culturally sensitive manner. The interview guide was developed by mapping the research questions and reviewing relevant literature. To ensure content validity, the guide was reviewed by a panel of experts before being piloted. The interview guide was organized into four thematic areas: (1) opening questions, (2) access and utilization of healthcare, (3) access barriers, and (4) adaptation strategies.

### 2.6 Data analysis

Thematic Analysis (TA) employed the Reflexive methodology developed by Braun and Clarke to interpret refugee perspectives and generate contextual meanings related to coping and adaptation experiences [22]. This approach embraces subjectivity, self-awareness, and critical reflection of the researcher, and it involves knowledge generation and participants as active contributors to this generation [23]. Through reflexivity, the authors critically assessed and reflected on their experiences, values, and assumptions in a way that ensures rigorous findings [24].

All coding was conducted manually, beginning with repeated readings of the transcripts to develop an initial set of descriptive (semantic) codes. As the analysis progressed, coding moved toward more conceptual and latent interpretations, allowing deeper patterns, assumptions, and meanings to emerge. The thematic structure developed then reviewed by the thesis co-advisor, who provided feedback and supported further refinement. Any questions or uncertainties regarding code interpretation were resolved through discussion until consensus was reached.

## 3 Results

Among the interviewed households (HHs), 60% were male. The majority of HHs were of a nuclear structure (90%), with an average family size of 5.1 members. Most interviewees were aged between 45 and 59 years (45%) and 30 and 44 years (40%). Education levels were generally low: 20% were illiterate, and 70% had completed only primary school. Finally, most families (60%) reported arrival during the peak of the crisis, between 2012 and 2013 (Table 1).

**Table 1:** Demographic characteristics.

	Total (n = 20)	mean, %
**Total number of HHs enrolled**	20	–
**HH members enrolled**	102	–
**Gender of head of HH**		
Female	2	10%
Male	18	90%
**Gender of interviewee**		
Female	8	40%
Male	12	60%
**Age distribution of interviewee (n = 20)**		
18-29	2	10%
30-44	8	40%
45-59	9	45%
More than 60	1	5%
**Education level of interviewee (n = 20)**		
Primary school	14	70%
Secondary school	2	10%
University	0	0.0%
Illiterate	4	20%
**Family Type (n = 20)**		
Nuclear	18	90%
Extended	2	10%
**Family Size**		
**Average family size**	20	5.1
1-3	2	10%
4-6	10	50%
7-9	8	40%
**Year arrives in Jordan**		
Before 2011	1	5%
2011	1	5%
2012	3	15%
2013	9	45%
2014 and after	6	30%

A broad range of adaptation strategies generated from the interviews. Refugees adopted various strategies based on their experience with the local healthcare system and their endogenous capacities. These adaptation strategies ranged from extremely negative to more constructive strategies. The thematic analysis identified ten distinct domains of adaptation strategies, varied from mechanisms directly related to health needs to broader livelihood-driven adaptations that indirectly influenced healthcare-seeking behavior. Ten key domains of adaptation developed and have been grouped into health-related strategies and non-health-related strategies.

## 4 Health-Related adaptation strategies

### 4.1 Self-medication

Self-medication, or the irrational use of healthcare is practiced when the patient uses medication without undergoing proper medical evaluation, and complete clinical examination or investigation. This practice appeared as a common theme, reported by nine families, who were aiming to reduce healthcare costs by avoiding consultation fees, and in some cases, the cost of investigations. A caregiving mother described her experience with healthcare as the following:

“*For private care, it’s available but needs money, I don’t have money, if I have enough money, I will treat her in private clinics. There are competent doctors, and Jordan is well known having the best doctors and best treatment, but it needs money and its high cost, I don’t have. I gain 200 to 300 Jordanian Dinars (JoDs, 1 JoD = 0.708 USD) a month, how I can treat my children if they have flu? I go to the pharmacy directly and not to the doctor in order not to pay doctor fees, I pay for medicine only*” (Jhad, 36)

### 4.2 Use alternative medicine

Five interviewees reported using alternative medicine as an adaptation strategy to reduce healthcare costs or to postpone seeking care. Two main approaches were adopted under this domain; some participants relied on painkillers to delay the need for professional care or to ease health problems, while others tried traditional herbs to solve their health complaints. A mother stated:

“*In such time of the year, you feel there are a lot of diseases, I mean there are viruses in the air because of low raining rate -may God bless us-, I might need antibiotic syrup but because there is no income, I use things available at home (natural) and try to treat by it*” (Zahra, 43).

Another 50 years old female was asked on times when she or her family needed healthcare and was not able to grant, she said:

“*many occasions happened this year but didn’t go, I took analgesics and don’t go*” (Amal, 50)

### 4.3 Delay seeking care

Delaying healthcare was used as an adaptation strategy by four families who lacked the financial capacity to seek timely medical attention. The duration of these delays varied depending on the seriousness of the health problem and the family’s perception of the needed healthcare. Few families postpended the care until it escalated to an emergency status, while others skipped visits for a chronic condition, or delayed it until they could secure additional support. One participant living with multiple chronic conditions said:

“*When we need healthcare, we wait until it gets worst, may some good people help us and send me to a hospital when I reach a point where I need hospital care*” (Ameera, 46)

### 4.4 Stop or reduce medication intake

Reducing or stopping medication intake was identified as a strategy among individuals suffering from chronic conditions that require regular care and medication. Two participants adopted this strategy as a way to meet their healthcare needs while reasonably reducing healthcare costs. One such participant, a 50-year-old mother with multiple chronic diseases, including respiratory condition, said:

“*I have chronic bronchitis; it impacts my chest. I can’t breathe and found to have shortage in oxygen and vitamin deficiency. He told me (the doctor) according to the investigation that I suffer from lung rheumatism, I went there one time then I stopped because of financial depletion and bad situation. This inhaler (medication), I have to take and not to stop at all as it cleans the secretions. Honestly, I ceased it, it’s my hope…, I ceased it, from where I can get money*” (Anwar, 50)

### 4.5 Pursue free services

Pursuing free services appeared as recurring behavior during the interviews. Several participants stated this as an adaptation strategy, particularly when left with limited financial capacity or a lack of viable alternatives. Six participants specifically mentioned relying on NGO- provided care, which is offered on a free basis, to meet their health needs. A 38-year-old mother required a radiological investigation that cost about 100 JoDs. She delayed the procedure for an extended period and avoided borrowing money as she was afraid about her ability to reconcile or the risk of falling into debt. When asked about the strategies she adopted to get the needed service, she said:

“*I have to apply for a center to support and wait until my turn comes. However, even if I get 50% discount, I will not be able to pay and make it*” (Smeera, 38).

## 5 Non-health-related adaptation strategies

### 5.1 Borrow money

Borrowing money or using savings was primarily utilized by nearly half of the families to meet their livelihood needs, including healthcare. Eleven interviewees reported borrowing money to cover their basic needs, with eight clearly citing emergency healthcare or access to chronic disease treatment as the primary reason. When asked about times she was unable to pay for needed services, one mother said:

“*sometimes needs are raised up, and we are obliged to meet; something that breaks the back but nothing is available (money). For anything urgent, I have to go and borrow money*” (Reham, 37).

### 5.2 Prioritize between health and other livelihood needs

Identification of priorities for families was a significant task for the head of household and generated as a main domain in eleven interviews. Participants described several reprioritization strategies used to balance health needs with other livelihood demands. These strategies aimed to maintain balance and meet the basics for their families. Reprioritization occurred into two directions: some families prioritize health over other needs, while others reprioritize other needs over health such as food, clothing, sanitation materials, and learning materials.

The modes of prioritization included redirecting other forms of livelihood assistance (e.g., monthly cash assistance or food coupons) to cover health needs, cancelling or reducing the consumption of essential goods, opting for lower quality food or products, or selling home assets to fund healthcare. A mother of five children said:

“*It reduces nutrition especially after suspension of monthly assistances (food coupon) I get from United Nation, it will be utilized for many other things, and I will not keep it only for food”.* Another mother of three minors said*: “frankly, I might select anything in the week when we buy medication for my daughter, we eat anything in order to meet our needs*” (Smeera, 38).

### 5.3 Collect donations

Collecting donations was mentioned as an interim strategy to meet healthcare needs when no other options were available or when refugees had fallen into debt due to catastrophic health expenditure. For those who relied on donation collection, this strategy was used either to secure unaffordable medication or to obtain them through in-kind donation. One chronically ill female refugee, who suffers from hypertension, rheumatoid disease, and vascular disease needs several medications that were not affordable, described her experience as follows:

“*I go to a doctor I know him since a while, one of them is Syrian and he give me as a donation. Frankly, I can’t buy them as they are expensive. Sometimes, I got it from a neighbor, they have health insurance and sometimes they have the same hypertension medication I use, they give it to me if they have sufficient stock*” (Azhar, 56)

Another refugee family fell into debt due to catastrophic health expenditure due to a work related injury, said:

“*I have 2,200 JoDs debt, swear, I collect some from my brothers and neighborhoods*” (Halema, 28).

### 5.4 Child labor

Sending children to work was identified as an adaptation strategy by three families. Although child labor is considered a form of illegal work, these families reported resorting to it as a means to gain some income to meet the family’s basic needs, including healthcare. For instance, a 50-year-old mother, while describing her family’s life, said:

“*We live in a simple house with simple furniture that took us long time to secure, last piece I brought was refrigerator, I am UNHCR registered. My son work, he is the only one, he was in the school and was at higher rank with 86% averages, he left the school at 10th grade in order to work and meet our needs*” (Aisha, 50).

### 5.5 Onward movement

One participant talked about leaving for a third country as a solution for achieving better living conditions. This family was facing multiple livelihood challenges, including overwhelming debts from unpaid home rent and multiple health conditions that are not managed correctly. Reflecting on their situation, he said:

“*Frankly, I need to go outside, even if I live for two days. I am psychologically tired, I feel desperate, I heard from those who went out, if I leave there, I will receive financial support, they will treat me and others, I wish I can leave outside”*(Ali, 60).

## 6 Discussion

This study identified a wide range of adaptation strategies employed by Syrian refugees to navigate barriers to healthcare. These strategies fell into two overreaching categories: health related strategies, such as self-medication, delay-seeking care, discontinuing medication use, turning to alternative medicines, or searching for free providers; and non-health related strategies that indirectly supported access to healthcare, including reprioritization between essential needs, donation collection, or engaging in illegal income-generation such as child labor.

A clear link emerged between these adaptation strategies and rising healthcare costs, as most participants reported an increase in healthcare costs following changes introduced to the access policies and copayment schemes that applied during the period of data collection. Therefore, limited economic capacities significantly restricted refugees’ ability to access medical care, including doctor visits, obtaining medications, and medical procedures. This financial strain permeated other aspects of life needs such as shelter, food, education, and other aspects of livelihood.

Identifying these adaptation strategies was the central research question sought answer. However, existing literature on adaptation strategies among refugees over such protracted crises remains limited. For instance, an assessment carried out by the International Rescue Committee (IRC) in 2018 identified several adaptations, including use of over-the-counter medication, traditional medicine, home deliveries, borrowing money, reducing expenditure, or selling food coupons. These strategies were employed either to reduce expenditure on health or increase financial capacity of families to meet their health needs at minimal costs [25].

Findings from this qualitative assessment corroborate several of the IRC results, particularly the reliance on spending savings, borrowing money, pursue free services, reducing or discontinuing medication, and using alternative medicine. However, this study extends the existing evidence by identifying additional adaptation mechanisms not previously described in the IRC report. These newly observed strategies were consolidated into two main categories; health-related, and non-health related based on their impact. These new thematic adaptations included self-medication, delay seeking care, prioritizing between health and other livelihood needs, collect donation, child labor and onward movement

All these adaptation strategies were found to have negative and sometimes harmful effects on both health and other aspects of livelihood. These findings align with those reported by Nabulsi and his team, whose qualitative assessment among Syrian refugees in Lebanon revealed comparable adaptation mechanisms. Their study found engagement in informal employment, child labor, accruing debt and poor living conditions were utilized by refugees to meet their family needs, including healthcare [26].

The refugees typically have a low health promotion scale and poorer health outcomes compared to local nationals who benefit from established welfare systems [27]. Therefore, understanding how refugees respond when they face shortages in their means of livelihood including healthcare is critical,. However, attempts to identify the root causes of such behaviors may not always reveal a clear rationale or a direct connection between health and other livelihood shortages, which are difficult to isolate in qualitative inquiry.

Nevertheless, shortages in basic needs often drive families to adopt a range of interrelated adaptation strategies. These include deprioritizing certain needs in favor of others, relocating resources, or utilizing negative coping such as child labor. A common reason found across all areas of livelihoods, including health, was financial hardship. In a longitudinal study, Torlinska and her team investigated the correlation between financial hardship and refugees’ well-being, affirming that financial difficulties are associated with diminished physical and mental well-being [28].

Furthermore, service utilization among refugees is often compromised for several reasons [29], refugees tend to avoid public service when they feel they can manage the situation on their own [30]. This general approach toward service utilization in the country of asylum combined with social, cultural, economic, and structural factors contributes to the adaptation of several strategies.

The ongoing instability experienced by refugees, combined with fluctuating health policies, increases in healthcare cost, persistent economic hardship, and persistent sense of temporariness despite years of displacement often compels refugees to adopt short term, temporary solutions. While these conditions underscore the dynamic nature of adapted health policy, the structural and perceptual barriers driving adaptation strategies are likely to remain salient. Therefore, understanding refugees’ experiences in accessing healthcare, assessing access barriers, and recognizing their capacities and expectations is essential for interpreting their health-seeking behaviors and motivation for adaptations. Insight into these behaviors and adaptation strategies is key for structuring an efficient health intervention that addresses the root causes rather than only its impact, ultimately leading to improved and sustainable health outcomes. Essentially, this study contributes new depth by identifying several adaptation strategies not previously documented in earlier literature and by demonstrating how health-related and livelihood-related adaptations interact within the broader context of protracted crisis.

## 7 Conclusion and recommendation

When refugees face longstanding barriers to accessing healthcare or other basic needs, they adopt several strategies to cope. Some of these adaptation strategies can be considered non-harmful, such as pursuing free healthcare or collecting donations. However, many others are harmful or have an extended impact on overall well-being. These include self-medication, delay seeking care, discontinuing the use of medicine, and using alternative medicine. Additionally, certain strategies such as borrowing money, engaging in illegal labor, undertaking onward movement, or reprioritizing between health and other livelihood needs can have extended impact on other aspects of life.

Persistent exposure to shortages and barriers in accessing essential healthcare services and other basic livelihood needs can lead to complex and interrelated consequences for refugees. Over time, this may give rise to new and unpredictable behaviors. Many of these newly adopted strategies may reflect opposing behavioral patterns and may have an extended impact on refugees’ health stability and their ability to lead resilient lives. Moreover, these dynamics may also affect the hosting community, potentially escalating social tension between both communities.

Refugees’ healthcare access and utilization behaviors are multidimensional and dynamically changing over time [31]. Understanding these dynamics requires an integrated approach that considers contextual characteristics, followed by identifying health outlook and current needs, identifying seeking behaviors, recognizing access barriers, and analyzing adaptation strategies that emerge in response. It is also essential to assess how these factors interact and establish outcomes for both refugees and hosting communities. However, understanding the interrelationship between all health identifiers and other livelihoods remain underexplored and warrants more in-depth research and modelling. Additionally, the dynamic nature of this interrelation is significantly influenced by the evolving context of displacement and necessitates continuous monitoring to anticipate emerging adaptation patterns and their implications.

In conclusion, improving refugees’ access to healthcare is essential for reducing reliance on harmful adaptation strategies. Health authorities should lower out-of-pocket costs for essential care especially at the primary healthcare level to prevent delays in seeking treatment. Service providers should also simplify administrative procedures and support communication with communities to reduce confusion. Finally, coordinated monitoring systems across MoH and humanitarian actors are needed to detect emerging adaptation behaviors as policies and economic conditions shift.

## 8 Strengths and limitations

This study contributes significantly to the discourse on health system inequality in the field of refugee health. One of its key strengths lies in the use of purposive sampling among a hard-to-reach population, as well as the application of reflexive thematic analysis, which strengthens the context-driven conclusions.

However, some limitations arose due to several contextual factors. Ethical considerations arose during data collection, particularly related to participants’ vulnerability and the primary investigator’s dual role as a humanitarian worker. This dynamic may have impacted the consent process, given the increased vulnerability of participants and potential expectations. The sample was selected from three community centers primarily receiving urban refugees in two governorates. While the limited geographical scope may have constrained the potential for maximum variation, selecting community centers rather than healthcare facilities enhanced the diversity of participants and supported the identification of key targeted variables related to health-seeking behaviors and adaptation strategies.

The interrelated nature of refugees’ vulnerabilities, including health vulnerability, poses challenges in understanding and interpreting how these overlapping factors accumulate and interact. Additionally, the study partially captured facilitators to access health, which may have influenced the understanding of perceived needs and adaptation strategies.

Moreover, the inability to establish clear causal relationships represents an important analytical limitation. Although the study identified patterns such as limited service utilization associated with perceived discrimination, access challenges, and economic barriers, these relationships remain hypothesized rather than confirmed due to the qualitative design. Ongoing instability and policy fluctuations during the data collection period may also have shaped participants’ narratives, adding more complexity to interpreting motivations behind specific strategies.

Finally, the sample was predominantly composed of male respondents. This gender imbalance may hinder obtaining the perceived needs and strategies of female household members.

## Supporting information

S1 Checklist Checklist. (DOCX)
